# ADAR1 function affects HPV replication and is associated to recurrent human papillomavirus-induced dysplasia in HIV coinfected individuals

**DOI:** 10.1038/s41598-019-56422-x

**Published:** 2019-12-27

**Authors:** Maria Pujantell, Roger Badia, Iván Galván-Femenía, Edurne Garcia-Vidal, Rafael de Cid, Carmen Alcalde, Antonio Tarrats, Marta Piñol, Francesc Garcia, Ana M. Chamorro, Boris Revollo, Sebastian Videla, David Parés, Javier Corral, Cristina Tural, Guillem Sirera, José A. Esté, Ester Ballana, Eva Riveira-Muñoz

**Affiliations:** 1 AIDS Research Institute-IrsiCaixa, Badalona, Spain; 2 Genomes for Life-GCAT Lab Group - Program of Predictive and Personalized Medicine of Cancer (PMPPC), Badalona, Spain; 3 Health Research Institute Germans Trias i Pujol (IGTP), Badalona, Spain; 4 Fundació Lluita contra la Sida, Hospital Germans Trias i Pujol, Badalona, Spain; 5 Department of Gynecology, Hospital Germans Trias i Pujol, Badalona, Spain; 6 Department of Surgery, Hospital Germans Trias i Pujol, Badalona, Spain; 7 Department of Internal Medicine, Hospital Germans Trias i Pujol, Badalona, Spain

**Keywords:** Haplotypes, Viral infection, Infection

## Abstract

Infection by human papillomavirus (HPV) alters the microenvironment of keratinocytes as a mechanism to evade the immune system. A-to-I editing by ADAR1 has been reported to regulate innate immunity in response to viral infections. Here, we evaluated the role of ADAR1 in HPV infection *in vitro* and *in vivo*. Innate immune activation was characterized in human keratinocyte cell lines constitutively infected or not with HPV. ADAR1 knockdown induced an innate immune response through enhanced expression of RIG-I-like receptors (RLR) signaling cascade, over-production of type-I IFNs and pro-inflammatory cytokines. ADAR1 knockdown enhanced expression of HPV proteins, a process dependent on innate immune function as no A-to-I editing could be identified in HPV transcripts. A genetic association study was performed in a cohort of HPV/HIV infected individuals followed for a median of 6 years (range 0.1–24). We identified the low frequency haplotype AACCAT significantly associated with recurrent HPV dysplasia, suggesting a role of ADAR1 in the outcome of HPV infection in HIV+ individuals. In summary, our results suggest that ADAR1-mediated innate immune activation may influence HPV disease outcome, therefore indicating that modification of innate immune effectors regulated by ADAR1 could be a therapeutic strategy against HPV infection.

## Introduction

HPV is a small non-enveloped double-stranded DNA virus that infects keratinocytes. Most HPV infections clear spontaneously, however, persistent HPV infection is strongly associated with risk of several cancers including cervical, laryngeal, esophageal and anal cancer^1^. In immunosuppressed individuals there is a higher rate of cervical and anal HPV infections^2^. HPV infection favors HIV acquisition, and HIV-infected individuals encompass a heavier burden of HPV-induced dysplasia and cancer due to progressive immune suppression and impaired cell-mediated immunity. Persistent HPV infections are strong determinants of anal high-grade squamous intraepithelial lesions (HSIL)^3^.

Innate immune responses are characterized by production of a complex network of cytokines that effectively activate cellular immune cells^4^. Pattern recognition receptors (PRRs) include the RNA-binding helicase family of RIG-I-like receptors (RLR: RIG-I and MDA5), and the family of Toll-like receptors (TLR). In response to viral infections, produced type I interferons (IFN) activate the Janus kinase-signal transducer and activator of transcription (JAK-STAT) signaling pathway, which, in turn, induces the expression of additional interferon signaling genes (ISG) with direct antiviral properties^5^. HPV has developed mechanisms to evade the host surveillance by suppressing inflammatory immune pathways, by downregulating IFN response or through decreased expression of PRRs^6^.

ADAR1 is an adenosine deaminase that converts adenosines to inosines (A-to-I editing) in double stranded RNA (dsRNA). Inosine is recognized as guanosine by splicing and translational machineries, therefore A-to-I editing may lead to changes in coding and amino acid sequences^7^. ADAR1 has been found to regulate innate immune responses and the IFN-signaling pathway^8–10^. Loss of ADAR1 function causes a severe autoimmune disease, as A-to-I editing is an intracellular mechanism that distinguishes cellular versus pathogen-derived nucleic acids^11^.

Importantly, there is strong evidence indicating that both catalytic and non-enzymatic functions of ADAR1 may participate in a number of malignant diseases^12^, including chronic myeloid leukemia^13^, hepatocellular carcinoma^14^, esophageal squamous cell carcinoma^15^ and cervical cancer progression^16^. A-to-I editing of dsRNA has been linked to the replication process of different viruses, being ADAR1 function associated to enhanced or reduced viral growth or persistence^17^. ADAR1 is a proviral factor In HIV-1 infection, *i*.*e*. ADAR1 expression is linked to enhanced viral replication^10,18,19^. Conversely, ADAR1 appears to prevent HCV replication^20–22^ and has been associated to HCV-associated liver fibrosis^23^. The role of ADAR1 on HPV infection and oncogenicity has not been explored.

Here, we have investigated the role of ADAR1 as a regulator of innate and antiviral immune function in HPV-infected cells and in a cohort of HPV/HIV coinfected individuals. We show that depletion of ADAR1 in keratinocytes alters RLR, IFN and ISG expression and leads to increased HPV expression in cell culture. Moreover, genetic association studies demonstrate that an *ADAR1* haplotype was associated to recurrent relapse in HPV-associated dysplasia in a cohort of HPV/HIV co-infected subjects.

## Results

### ADAR1 regulates type I IFN and innate immune activation in keratinocytes cell lines

Protein expression of innate immune effectors was first characterized in two keratinocyte cell lines SiHa HPV16+ and HaCaT HPV- (Supplementary Fig. 1). ADAR1 and MDA5 protein expression is similar between the two cell lines, however SiHa cells have higher RIG-I and phosphorylated STAT1 protein expression compared to HaCaT cells. As expected, expression of oncoprotein HPV16 E7 was only present in SiHa cells.

**Figure 1:**
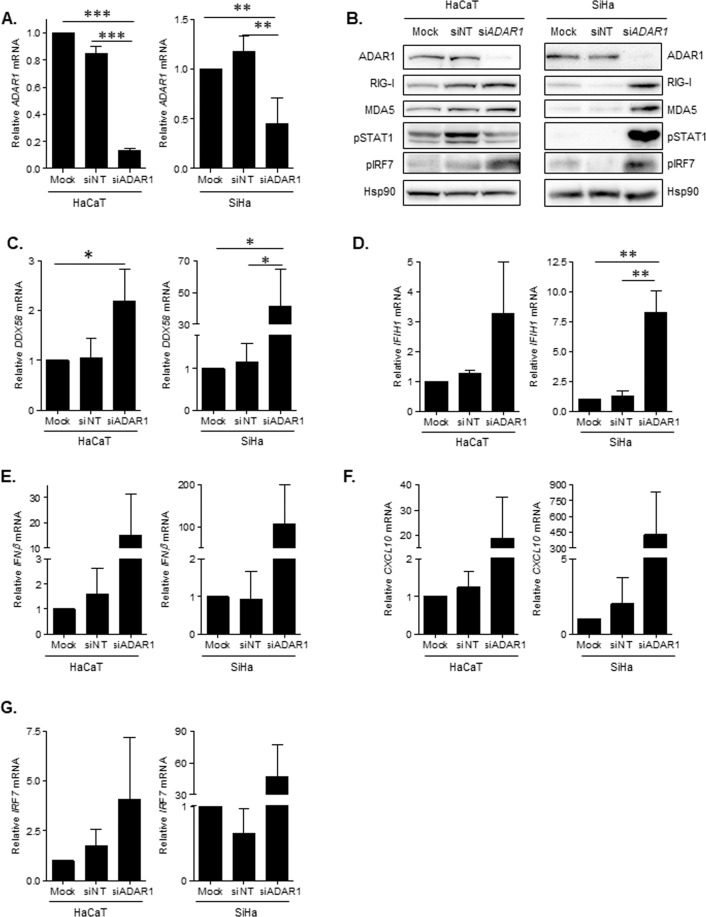
Characterization of ADAR1 knockdown profile in HaCaT and SiHa cell line. (**A**) Relative mRNA of *ADAR1* measured by quantitative PCR. ADAR1 expression was significantly downregulated in siADAR1, whereas expression level did not change in siNT. (**B**) Protein expression of innate immune factors involved in sensing of nucleic acids and IFN-I response in siRNA-treated cells. Downregulation of ADAR1p150 correlates with upregulation of RIG-I and MDA5, increased phosphorylation of STAT1 and transcriptional factor IRF7 in HaCaT and SiHa cell line. A representative Western blot is shown. The figure shows the cropped gels/blots obtained by each protein evaluation. Full-length blots of each tested protein are included in supplementary material. (**C**) mRNA expression of *DDX58* in siRNA-treated cells measured by qPCR. *DDX58* expression was significantly upregulated in siADAR1, whereas expression level did not change in siNT. (**D**) Relative mRNA expression of *IFIH1* in siRNA-treated HaCaT and SiHa cells measured by quantitative PCR and normalized to GAPDH expression. *IFIH1* gene expression was upregulated in siADAR1, whereas expression level in siNT did not change in both cell lines. (**E**) Relative mRNA expression of *IFNB1* in siRNA-treated cells measured by qPCR and normalized to GAPDH expression. *IFNB1* gene expression was upregulated in siADAR1, whereas expression level in siNT did not change in any cell line. (**F**) Relative mRNA expression of *CXCL10* in siRNA-treated cells measured by qPCR and normalized to GAPDH expression. *CXCL10* gene expression was upregulated in siADAR1, whereas expression level in siNT did not change in both cell lines. (**G**) Relative mRNA expression of *IRF7* in siRNA-treated cells measured by qPCR and normalized to GAPDH expression. *IRF7* gene expression was upregulated in siADAR1, whereas expression level in siNT did not change in both cell lines. Left side graphs represent HaCaT and right side SiHa cell line. Data represents mean ± SD of at least 5 independent experiments and is normalized to Mock-transfected SiHa or HaCaT cells. *p < 0.05; **p < 0.005; ***p < 0.0005.

To investigate the role of ADAR1 in cells susceptible to HPV infection, ADAR1 was downregulated in both keratinocytes cell lines using RNA interference as an *in vitro* model for evaluating its contribution to HPV replication. In both cell lines, ADAR1 expression was significantly downregulated at the mRNA and protein level (Fig. 1A, p = 0.0001 and Fig. 1B ADAR1). Interestingly, ADAR1 downregulation led to a significant increase in *DDX58* (Fig. 1C, p = 0.0133) and *IFIH1* (Fig. 1D, p = 0.0024) mRNA, as well as protein expression (Fig. 1B, RIG-I and MDA5, respectively). Furthermore, ADAR1 downregulation induced the expression of *IFNB1* (Fig. 1E) and the ISG *CXCL10* (Fig. 1F). Accordingly, siRNA-ADAR1 overexpressed transcriptional factor IRF7 at the mRNA level (Fig. 1G) and increased phosphorylation of IRF7 and STAT1 as seen by Western blot (Fig. 1B, pIRF7 and pSTAT1), indicating activation of the innate immune response. Importantly, confirmatory siRNA sequences targeting ADAR1 showed similar effects in activation of type I IFN response (Supplementary Fig. 2). To further confirm the link between ADAR1 and RIG-I function, depletion of ADAR1 in a cell model harboring a signaling-incompetent endogenous RIG-I was performed. Interestingly, ADAR1 downregulation only led to increased IFN production in the presence of a functional RIG-I (Supplementary Fig. 3). These data are suggestive of a role of ADAR1 as a negative regulator of innate immune response in keratinocytes cell lines.

### ADAR1 knockdown in SiHa cells induces a pro-inflammatory phenotype

**Figure 2:**
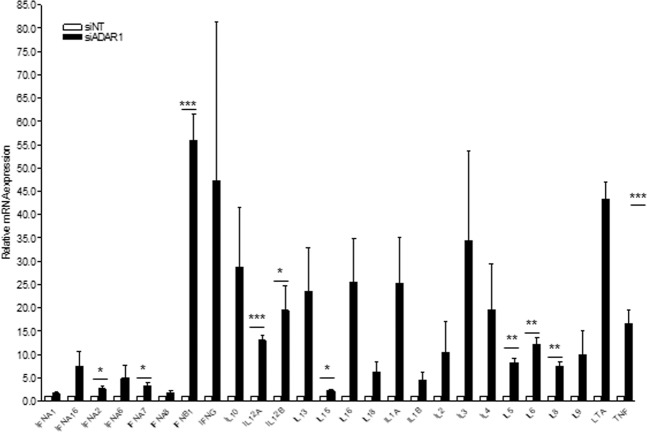
Cytokine induction in treated siRNA-ADAR1 SiHa (HPV+) cells. (**A**) Cytokine expression profile of ADAR1 knockdown in SiHa cell line. Relative mRNA expression of the different cytokines normalized to siNT-transfected cells is depicted. Data represents mean ± SD of 3 different experiments. *p < 0.05; **p < 0.005; ***p < 0.0005.

To understand the microenvironment induced by ADAR1 knockdown, cytokine production was evaluated in HPV16 + SiHa cells, which harbor integrated copies of HPV. Significant upregulation of nine different IFNs and cytokine genes were observed in siRNA-ADAR1 cells (*IFNA2* (p = 0.0341); *IFNA7* (p = 0.0365); *IFNB1* (p = 0.0006); *IL12A* (p = 0.0005); *IL12B* (p = 0.0252); *IL15* (p = 0.0292); *IL5* (p = 0.0011); *IL6* (p = 0.0015); *IL8* (p = 0.0025); LTA (p = 0.0004)) compared to siRNA-NT (Fig. 2). These results suggest ADAR1 knockdown is inducing a pro-inflammatory phenotype, not limited to the increase in IFN, and a significant innate immune activation that may alter the cell microenvironment and their response to viral infection.

### ADAR1 regulates HPV expression in SiHa cell line

**Figure 3:**
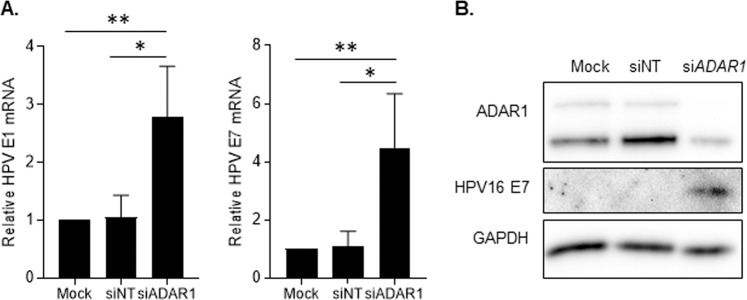
ADAR1 knockdown induces HPV16 expression in SiHa cell line. (**A**) Relative expression of *HPV16 E1* and *HPV16 E7* mRNA in siRNA-treated SiHa cells measured by quantitative PCR and normalized to GAPDH expression. ADAR1 downregulation induces 2.7-fold *HPV16 E1* expression and 4.4-fold *HPV16 E7* expression compared to Mock transfected SiHa cells. (**B**) Protein expression of ADAR1 and HPV E7 in siRNA-treated SiHa cells. Downregulation of ADAR1 in SiHa cell line induces HPV16 E7 protein expression. GAPDH was used as loading control. A representative western blot is shown. The figure shows the cropped gels/blots obtained by each protein evaluation. Full-length blots of each tested protein are included in supplementary material. *p < 0.05; **p < 0.005.

The role of ADAR1 on HPV replication was evaluated in HPV16 + SiHa cells, containing the HPV genome integrated in chromosome 13q21^24^. HPV viral RNA and protein expression was measured in an ADAR1 loss-of-function cell model. Interestingly, downregulation of ADAR1 significantly upregulated mRNA expression of *HPV16 E1* and *HPV16 E7* (Fig. 3A, p = 0.0068 and p = 0.0098, respectively). Similarly, HPV16 E7 protein expression was also increased, as measured by Western blot (Fig. 3B, HPV16 E7). As expected, siRNA-NT did not have any effect on HPV mRNA nor protein expression in SiHa cells (Fig. 3A,B).

Transfection of SiHa cells with PolyI:C, a potent inducer of innate immune activation, enhanced also HPV viral RNA expression, indicating that ADAR1 innate immune activation might explain enhanced HPV replication (Supplementary Fig. 4). To further delineate the pathway underlying ADAR1-mediated regulation of HPV infection, RLR expression was knockdown in wild-type or ADAR1-depleted SiHa cells. As expected, RIG-I and MDA5 knockdown did neither increase innate immunity nor HPV transcription in ADAR1-expressing cells (Supplementary Fig. 5). Importantly, ADAR1-mediated induction of innate immunity was limited in RIG-I or MDA5-depleted cells, suggesting that ADAR1 effect is dependent on RLR function. Overall, these results suggest an innate immune mediated antiviral role of ADAR1 function in HPV infection *in vitro*.

### A-to-I editing by ADAR1 is not present in HPV transcripts in SiHa cell line

**Figure 4:**
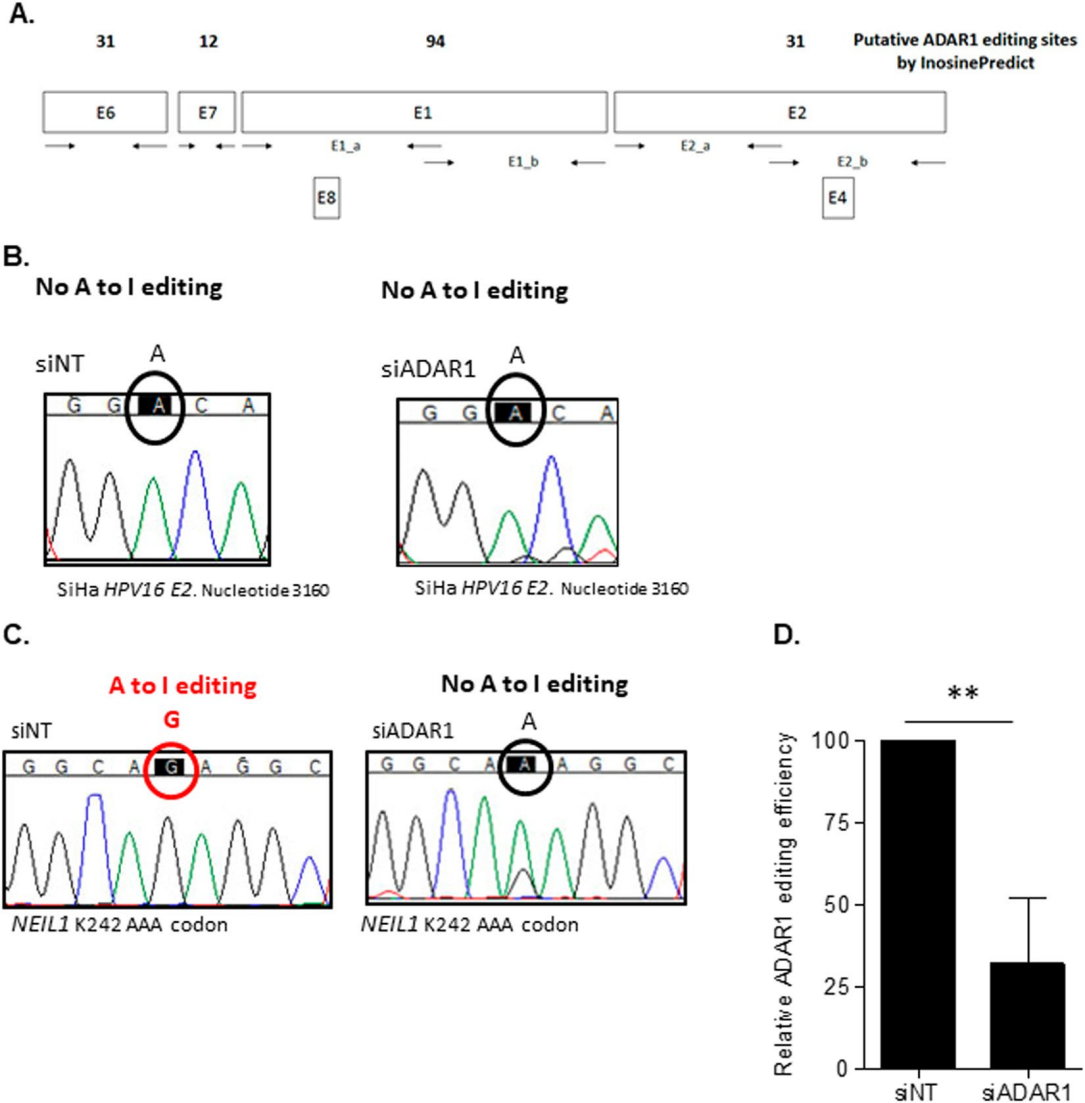
Putative ADAR1-mediated A-to-I editing sites in HPV16 and NEIL1 transcripts. (**A**) Schematic representation of designed primers and putative A-to-I editing sites of HPV16 integrated sequence in SiHa cell line. InosinePredict was used to estimate site and number of ADAR1 A-to-I editing sites. (**B**) Representative DNA sequencing chromatogram of *HPV16 E2* putative editing site with no A-to-I editing detected in nucleotide 3160 in SiHa-derived transcripts. HPV16 SiHa transcripts were amplified from RQ-PCR using primers represented in (**A**), sequenced and aligned siNT and siADAR1 sequences to detect A-to-I editing events. siNT and siADAR1 chromatograms are from the same experiment. (**C**) Representative DNA sequencing chromatogram of *NEIL1* transcripts showing A-to-I editing at known K242 codon in siNT, but not in siADAR1 SiHa transcripts. A-to-I editing function is confirmed in siNT SiHa transcripts. siNT and siADAR1 chromatograms are from the same experiment. (**D**) Relative ADAR1 editing efficiency in SiHa *NEIL1* transcript. Reduced edited peak in siADAR1 was relativized to edited peak in siNT. Edited adenosines to inosines are detected as Gs by direct sequencing. Data represents mean ± SD of at least 3 independent experiments. A representative chromatograms is shown. **p < 0.005.

To determine the role of ADAR1-mediated-A-to-I editing involved in the regulation of HPV expression, we evaluated A-to-I editing sites in HPV16 transcripts from SiHa cell line. InosinePredict software was used to estimate putative A-to-I editing sites and primers were designed to amplify HPV16 transcript fragments (Fig. 4A). 148 putative ADAR1 modification sites were estimated (Fig. 4A). HPV16 transcripts were amplified, sequenced and scanned for base changes that could be the result of adenosine deamination in mRNA. Although all detectable transcripts in SiHa cells were amplified and scanned, no A-to-I differences were found (Fig. 4B).

As a control, we evaluated A-to-I editing in the known ADAR1-target *NEIL1*. As expected, A-to-I editing was present in *NEIL1*, whereas in siRNA-ADAR1 samples the site was not edited (Fig. 4C). Moreover, we determined the relative A-to-I editing efficiency at the *NEIL1* site, ADAR1 knockdown cells showed a significant reduction of approximately 70% inhibition (Fig. 4D, p = 0.0043), confirming editing function of ADAR1 is decreased in ADAR1 knockdown cells.

In summary, our results suggest that ADAR1 effect over HPV infection is not determined by direct A-to-I editing of HPV genome.

### The ADAR1 haplotype AACCAT is associated with recurrent dysplasia in HPV patients

To study the effect of ADAR1 in the development of HPV infection, a genetic association study was designed using a cohort of HPV/HIV coinfected individuals (n = 173). Six ADAR1 polymorphisms covering all known genetic variation in *ADAR1* gene (rs6699729, rs3766927, rs3766925, rs3766924, rs9616 and rs9427097) were genotyped and tested for association to recurrence of HPV associated dysplasia (relapse in HPV associated dysplasia). Selected demographic and clinical information are summarized in Table 1. Genetic association of each SNP was adjusted by age, sex, time since first cytology and HIV infection time, CD4 nadir, and oncogenic HPV genotypes in a logistic regression model. When testing for single SNP-association, no significant association was found to HPV recurrence (p-values > 0.05).

**Table 1:** Clinical characteristics of HPV/HIV-1 coinfected patients.

	All	Recurrent dysplasia	Non- Recurrent dysplasia	p
Individuals, n (%, CI)	173 (100)	52 (30%, **95% CI: 24–37%**)	121 (70%, **95% CI: 63–76%**)	—
Gender, female/male (% of total)	32 (18.5)/141 (81.5)	10 (19)/42 (81)	22 (18)/99 (82)	NS
Age, years median (range)	45 (23–77)	46 (24–77)	43 (23–52)	NS
Follow-up time since first cytology, median (years)	5.8	5.6	6.1	NS
Time to first dysplasia, median years (range)	2.6 (0.7–10)	1.6 (0.7–10)	2.2 (0.1–6.9)	NS
Time since first visit to HIV clinic (years)	12.8	10.7	13.3	NS
HPV genotypes, n				
Low oncogenic type		0	13	—
High oncogenic type		52	108	—
CD4 Nadir, median (range)	240 (22–993)	225 (2–576)	248 (80–993)	NS

CI: confidence interval, NS: not significant after Student’s T test. Time to first dysplasia calculated from the time to first cytology. Samples were considered high oncogenic type with one or more high risk oncogenic HPV types detected.

To further explore whether ADAR1 may influence recurrence in HPV-induced dysplasia, estimation of haplotype frequencies were performed. Haplotype-based analysis represent a valuable resource for investigating the genetic basis of disease susceptibility, albeit the identification of the functional variant has proven challenging. When we estimated ADAR1 haplotypes to HPV-dysplasia recurrence, we found the low frequency haplotype AACCAT was significantly associated with recurrent HPV dysplasia (2% and 8% of non-recurrent and recurrent HPV dysplasia respectively) (p_sim_ = 0.0064) (Table 2), suggesting that ADAR1 plays a role in HPV infection *in vivo*. Importantly, the number of malignancy-associated HPV types identified was not significantly different (p > 0.05) between groups of recurrent and non-recurrent HPV-associated dysplasia, indicating that ADAR1 was an independent factor from higher prevalence of oncogenic HPV types.

**Table 2:** Haplotype association results for recurrent dysplasia adjusted by sex, age, HIV infection, CD4 nadir and HPV oncogenic types.

Haplotype						Frequency			P-value	P-value_sim
rs6699729	rs3766925	rs3766927	rs3766924	rs9616	rs9427097	All	Non-recurrent dysplasia	Recurrent dysplasia		
A	A	C	C	A	T	0.041	0.022	0.081	**0.0084**	**0.0064**
A	A	T	C	A	T	0.249	0.271	0.204	0.075	0.084
T	T	C	C	A	G	0.139	0.132	0.151	0.336	0.338
A	T	T	C	A	T	0.069	0.061	0.089	0.349	0.361
A	T	C	C	A	G	0.015	0.019	0.008	0.525	0.592
T	T	C	C	T	T	0.252	0.256	0.24	0.647	0.656
A	T	C	T	A	T	0.177	0.179	0.177	0.879	0.886

Haplotype frequencies are shown for all the patients and for dysplasia groups.

*P-value_sim: corrected p-value after 10,000 permutations in the score test implemented in haplo.stats R package^44^.

## Discussion

HPV infection has been associated to suppressed immune factors, including cytokines and chemokines to evade immune detection. Genetic polymorphism in inflammatory pathways have been shown to constitute a risk in cervical cancer development in HPV infections^25^. We have evaluated the effect of ADAR1, a regulator of type-I IFN production and immune regulator^10^, in the context of HPV infection, including a cohort of HPV/HIV coinfected individuals. We show that ADAR1 may play a role in HPV infection by regulating the innate immune response.

HPV virus life cycle is tightly linked to cell differentiation. Infected SiHa cells have an active RLR signaling pathway and display type-I IFN activation compared to uninfected HaCaT keratinocytes. Signaling pathways leading to cell activation, proliferation and differentiation may promote HPV protein expression from integrated genomes that further drive cell differentiation and oncogenesis^26^. HPV viral protein E6 expression affects RLR signaling as well as other ISGs through an IFN-RLR-mediated induction^27^, as we observed by increased RIG-I expression and enhanced phosphorylation of STAT1. Thus, it would appear that innate immune activation of cells harboring integrated virus favors virus replication and disease progression. Nevertheless, this comparison is an initial indication that HPV gene and protein expression could affect key innate immune modulators, pivotal to the innate immune response.

A STING-activating nanovaccine boosts anti-tumor immunity in cancer immunotherapy^28^ and is able to inhibit expression of HPV E6/E7 in a tumor model, indicating that immune strategies targeting sensing of viral nucleic acids may be effective tumor growth inhibitors. Therefore, targeting ADAR1, which alters RNA sensing, RLR/MAVS, type-I IFN-mediated responses and it is upstream RIG-I signaling pathway could be a putative strategy to induce immune protection in keratinocytes. Here, we show that ADAR1 knockdown triggers immune and IFN type-I signaling activation, as previously reported in different cell lines and mouse models. We show that in either uninfected or infected keratinocytes, ADAR1 knockdown regulates expression of RLR sensors RIG-I and MDA5, phosphorylation of transcriptional factor IRF7, expression of IFN-mediated effectors as IFN-β and CXCL10 and enhanced phosphorylation of STAT1, a marker for type-I IFN activation. Furthermore, ADAR1 knockdown in infected keratinocytes induces a pro-inflammatory phenotype, similar to innate immune activation features observed in ADAR1-associated Aircadi-Goutieres syndrome patients and in KO mouse models^29,30^. In concordance with previous data, our study confirms the role of ADAR1 as a regulator of the IFN-signaling cascade and regulator of innate immune response also in keratinocytes^10,20^.

Even though HPV infected and uninfected ADAR1 knockdown keratinocytes display a similar innate immune signaling signature, ADAR1 knockdown in infected keratinocytes increases HPV viral expression. The link between RNA editing and HPV viral replication is not new. Previous evidence showing upregulation of immune signaling pathways has been associated to the cytidine deaminase APOBEC derived modifications^31^, suggesting that epitranscriptomic changes induced modulate innate immunity affecting and affect HPV outcome. Contrary to results showing that modulation of innate immune response is a consequence of the direct modification of HPV genome by APOBEC or ADAR1 in the case of other infections^7^, we did not detect any ADAR1-mediated A-to-I editing in HPV16 transcripts, therefore indicating that it might be related to the role of ADAR1 as a regulator of innate immune activation or to alternative functions of ADAR1.

Furthermore, we evaluated the genetic variation of ADAR1 in a cohort of HPV/HIV coinfected individuals and found a low frequency haplotype associated with recurrent relapse in HPV dysplasia, suggesting the putative role of ADAR1 in HPV outcome. Genetic association studies aim to identify the putative link of a given gene to a certain phenotype without informing on the specific molecular determinants of the association, thus, further studies are needed to better delineate the exact contribution of ADAR1 function to HPV recurrence. It is important to note that in our cohort, only a few individuals carry the associated haplotype, indicative of incomplete penetrance. Thus, the use of a haplotype-based analysis is able to better capture the whole genetic heterogeneity underlying HPV-associated dysplasia recurrence, but fail to identify the functional haplotype, therefore impeding to exclude the existence of additional factors that may affect HPV recurrence. In addition, whether differences in ADAR1 expression accounts for the effect seen in HPV-associated dysplasia cannot be rule out from our study. Finally, it has to be taken into account that from our data we cannot exclude that HIV infection might have a role in the genetic association found, and thus, data from additional cohorts infected only with HPV might solve this issue.

Our data is in concordance with the notion that deregulation of IFN production and inflammation-related genes are disadvantageous for effective HPV clearance and relapse dysplasia, also confirmed by the reported genetic association between inflammation-related genes and increased risk of cervical cancer development in women infected by HPV^25^. Therefore, we propose that ADAR1 could represent a key modifier of innate immune response to HPV infection and thus, its function can alter the development of oncogenic malignancies associated to HPV due to deregulation of RIG-I-mediated signaling.

Upon closer inspection of the haplotype analysis through the Genotype - Tissue Expression (GTEx) Project (GTEx Analysis Release V7) (https://gtexportal.org/home/gene/ADAR), we noticed that the associated haplotype only differs in one SNP from the most frequent ADAR1 haplotype^32^. SNP rs3766927 correlates with the expression of ADAR1, being the allele in the identified haplotype associated negatively with ADAR1 expression (β = −0.082, p = 0.0000092). Further investigation into the role of this haplotype would improve the significance of our findings. On the other hand, ADAR1 overexpression has been linked to better prognosis in individuals with oral squamous cells carcinoma^33^ and cervical cancer progression and angiogenesis^16^, providing further support to our data and pointing towards the involvement of epitranscriptomic modifications as determinants of HPV-associated pathogenesis. Importantly, loss of ADAR1 in tumors overcomes resistance to immune checkpoint blockade^34^, suggesting a potential benefit of immune checkpoint inhibitors in HPV infection.

In conclusion, we show that ADAR1 regulation of IFN type-I mediated responses, as well as regulation of RLR signaling pathway and induction of a pro-inflammatory phenotype, influence HPV expression. Lack of ADAR1 induces IFN production that in turn activates inflammation related genes and activates an innate immune response, which increases HPV expression. Induction of ADAR1 and regulation of its downstream effectors could be a therapeutic strategy against chronic HPV infection and disease.

## Materials and Methods

### Cells

HPV + SiHa cells^24^ were kindly provided by Dr. Berdasco Menendez from Bellvitge Biomedical Research Institute (IDIBELL, Spain). HaCaT cells (HPV16 negative) were obtained from Eucellbank (Banco de Células Eucariotas Celltech, UB). The hepatoma cell line Huh7 cells and its derivative Huh7.5 expressing RFP-NLS-IPS and a constitutive mitochondrial marker, mito-EGFP (was a king.pngt of Dr. Charles M. Rice (The Rockefeller University). Cells were cultured in Dulbecco’s modified Eagle’s medium (DMEM; Gibco, Madrid, Spain), supplemented with 10% heat-inactivated fetal calf serum (Gibco, Madrid, Spain), antibiotics (100 U/ml penicillin, 100 μg/ml streptomycin, Life Technologies) and maintained at 37 °C in a 5% CO_2_ incubator.

### Transfection and RNA interference

Cells were transfected using Lipofectamine 2000 (11668027, Invitrogen) in 24-well plate following manufacturer instructions for RNAi transfections as described previously^35^. Briefly, total of 50 pmol of the corresponding siRNA or mix of siRNA (25 pmol for each siRNA in double knockdown experiments) or 2 μg of Poly(I:C) was transfected in 1.25 10^5^ of the corresponding cell line. RNA and protein lysates were collected 64 h post-transfection for siRNA and 16 h post-transfection for Poly(I:C). siRNAs used for transfection were ON-TARGETplus Non-targeting siRNA Pool (D-001810-10), human ADAR1 siRNA-SMARTpool (L-008630-00), human IFIH1 siRNA-SMARTpool (L-013041-00), siGENOME human DDX58 siRNA-SMARTpool (M-012511-01) -and siGENOME human ADAR1 siRNA-SMARTpool (M-008630-01) all from Dharmacon, Waltham, USA.

### Quantitative RT-polymerase chain reaction (qRT-PCR)

Total RNA was extracted using the NucleoSpin RNA II kit (740955, Macherey-Nagel), as recommended by the manufacturer. Reverse transcription was performed using the PrimeScript™ RT-PCR Kit (RR036A, Takara) following manufacturer instructions. mRNA levels of all genes were measured by two-step quantitative RT-PCR and normalized to GAPDH mRNA expression using the DDCt method. Primers and DNA probes were TaqMan Gene expression assays from Life Technologies (TaqMan *ADAR1* Hs00241666, TaqMan *DDX58* Hs01061436, TaqMan *IFIH1* Hs01070321, TaqMan *CXCL10* Hs00171042, TaqMan *IFNB1A* Hs01077958, TaqMan *IRF7* Hs01014809, TaqMan *ISG15* Hs01921425, *HPV16-E1* Pa03453396) and previously described^36^ *HPV16-E7* primers and probe (forward primer 5′-AGAACCGGACAGAGCCCATTAC-3′, reverse primer 5′-GCCCATTAACAGGTCTTCCAAAG-3′ and probe 6FAM-CGCACAACCGAAGCGTAGAGTCACACTT-TAMRA).

### Human cytokine network array

Cytokine expression was evaluated by using the commercial TaqMan Human Cytokine Network array (4414255, Life Technologies). mRNA relative levels of cytokine genes were measured by two-step quantitative RT-PCR and normalized to GAPDH mRNA expression by using the DDCt method.

### Western blot

Protein expression was evaluated by Western blot as described before^37^. The following antibodies were used for immunoblotting: anti-rabbit and anti-mouse horseradish peroxidase-conjugated secondary antibodies (1:5000; Pierce); anti-human Hsp90 (1:1000; 610418, BD Biosciences), anti-ADAR1 (1:1000; 14175; Cell Signaling); anti-MDA5 (1:500; 5321; Cell Signaling); anti-RIG-I (1:1000; 3743; Cell Signaling); anti-phosphoSTAT1 (1:1000; 9167; Cell Signaling); anti-phosphorylated IRF7 (1:1000; 12390; Cell Signaling); anti-GAPDH (1:1000; ab9485; abcam); and anti-HPV16 E7 (1:1000; ab82601; Abcam).

### ADAR1 editing function

Total RNA was extracted and reversed transcribed as described above. Previously identified ADAR1 predicted modification sites in *NEIL1* transcripts were amplified as controls^10^, using the following primers: N1-TCOF 5′-TCCAGACCTGCTGGAGCTAT-3′, and N1-TCOR 5′-GGCCTTGGATTTCTTTTTG-3′. PCR product were then amplified using N1-TCIF 5′-CCCAAGGAAGTGGTCCAGTTGG-3′, and N1-TCIR 5′-CTGGAACCAGATGGTACGGCC-3′ for a nested amplification. PCR conditions were as follows 1 μL cDNA with thermocycler conditions of 98 °C for 30 s hot start, followed by 40 cycles of 98 °C for 30 s, 55 °C for 30 s, 72 °C for 30 s, and a final extension at 72 °C for 7 min. The nested PCR conditions with 1 μL of input PCR products were 98 °C for 30 s hot start, followed by 30 cycles of 95 °C for 10 s, 55 °C for 30 s, 72 °C for 30 s, and 72 °C for 7 min final extension, and were run on a 2% agarose gel to confirm band size of approximately 150 bp.

HPV primers were designed based on HPV16 genome sequence (GenBank accession number AF001599.1) using Primer3 software (*bioinfo*.*ut*.*ee/primer3-0*.*4*.*0/*). Primer pairs used for sequencing HPV transcripts expressed in SiHa cells were as follows: HPV16 E6 5′-TTCATGTATAAAACTAAGGGCGTAA-3′, 5′-CAGCTGGGTTTCTCTACGTGT-3′ (546 bp); HPV16 E7 5′-CATGGAGATACACCTACATTGC-3′, 5′-CTGAGAACAGATGGGGCACA-3′ (285 bp); HPV16 E1_a 5′-TGATCCTGCAGGTACCAATG-3′, 5′-TGCTGCCTTTGCATTACTAGTTT-3′ (613 bp); HPV16 E1_b 5′-GGGGAGAGGGTGTTAGTGAA-3′, 5′-CACATTGTTGCACAATCCTT-3′ (700 bp); HPV16 E2_a 5′-AGATGATGGAGGTGATTGGA-3′, 5′-CACATTTAAACGTTGGCAAAGA-3′ (623 bp); HPV16 E2_b 5′-CTCAAGGACGTGGTCCAGAT-3′, 5′-AGTAGACATACTGGGTTATCTGAGG-3′ (590 bp). PCR conditions for HPV16 transcripts were 95 °C for 5 min hot start, followed by 30 cycles of 95 °C for 30 s, 55 °C for 30 s, 72 °C for 30 s, and 72 °C for 5 min final extension. PCR products were run on a 2% agarose gel and different transcript bands were purified using QIAquick PCR purification kit (QIAGEN).

NEIL1 and HPV16 SiHa amplified products were treated with illustra™ ExoProStar™ (GEUS78210, Sigma) as described by the manufacturer. Samples were sequenced in a ABI3730XL system (Applied Biosystems).

Relative ADAR1 editing efficiency in *NEIL1* transcripts was estimated calculating the ratio of edited peak height (height of edited peak divided by the total height of edited and non-edited peak heights) for each sequence, and calculate the mean for the specific site for each independent experiment. Mean ratio of at least three independent experiments of siRNA-ADAR1 was then relativized to the mean ratio of at least three independent experiments of siRNA-NT.

### Patients and samples

173 HIV+ individuals were included in the study. This cohort is a single-center, prospective cohort of HIV-infected subjects annually assessed for HPV infection at anal, genital, and oral sites attending the Outpatient HIV Clinic of the Hospital Germans Trias i Pujol (Can Ruti Hospital, Badalona, Spain), as described elsewhere^38,39^. Clinical characteristics of patients are shown in Table 1.

The study was approved by the Ethics committee of Hospital Universitari Germans Trias i Pujol. Written informed consent was obtained from patients participating in the study. All methods were carried out in accordance with relevant guidelines and regulations and to the ethical principles suggested in the Declaration of Helsinki.

Individuals in the study were those suspected for anal intraepitlelial dysplasia (AID, n: 162) or cervical high-grade squamous intraepithelial lesions (HSIL, n: 11) following a cytology. If lesions were visualized, during high resolution anoscopy (HRA) or cervical colposcopy, a directed biopsy was performed for histological analysis. All patients with biopsy-proven of anal intraepithelial neoplasia-2 or -3 were treated with infrared ablation or surgery. A surgical resection (conization) was proposed in case of HSIL diagnosed confirmed by histology (CIN2 or CIN3). Cytologic changes were classified according to the Bethesda System: normal (no cell changes), atypical squamous cells of uncertain significance (ASCUS) or low- or high-grade squamous intraepithelial lesions (LSIL or HSIL, respectively). Overall recurrent dysplasia was defined as detection of at least two biopsy proven AIN 2-3 or CIN 2-3 at any time point during follow-up at the treated location or at new site^40^.

Anal samples were obtained introducing a cytobrush (Eurogine SL, Spain) 3 cm into the anal canal and gently rotating it for 30 to 45 seconds. The cytobrush was introduced into and shaken in a 20 mL of PreservCyt/Thin solution (Cytyc Iberia SL, Spain) for 30 seconds and assessed by the PAP method. Cervical citology samples were obtained from the exocervix with an Avre spatula and from the endocervix with a cotton swab and was assessed by the Pap method^41^. For HPV detection, the same samples were used to determine HPV genotype using the AnyplexTM II HPV28 real-time PCR (Seegene, Seoul, Korea) as described before^41^. HPV genotypes were classified as High (genotypes 16, 18, 31, 33, 35, 39, 45, 51, 52, 56, 58, 59, 66 and 68) and low (6, 11, 40, 42, 43, 44, 54, 61 and 70) oncogenic risk as described^41^.

### DNA extraction and SNP genotyping

Genomic DNA was extracted from anus or cervical samples^38^ using the QuickExtract™ DNA Extraction Solution 1.0 (QE09050, Epicentre Biotechnologies). Extracted DNA was used for each genotyping. Six ADAR1 SNPs (rs6699729, rs3766927, rs3766925, rs3766924, rs9616 and rs9427097) were selected based on linkage disequilibrium testing and cover all described variation in ADAR1 gene^23,42^. The variants were typed using TaqMan SNP genotyping assay (Assay num: C__30114879_10, C__11259682_10, C___222942_10, C__25800598_10, C___8724401_10 and C__303121822_10, respectively, Applied Biosystems) following manufacturer’s protocol. Reactions were analyzed on an ABI PRISM 7500 (Applied Biosystems) and allele calling was performed by AutoCaller Software v 1.1 (Applied Biosystems).

Genotyping data, minor allele frequency and Hardy-Weinberg equilibrium of selected SNPs are found in Supplementary Table 1.

### Statistical analysis

Experimental data are presented as mean ± SD. Paired Student’s t test was used for comparison between two groups, using the GraphPad Prism software. P-values lower than 0.05 were considered significant.

Genetic association analysis was computed using a logistic regression model^43^ for recurrent HPV dysplasia adjusted by age and sex, number of months since the first proctologist evaluation, CD4 nadir, number and type of HPV genotypes. We consider four inheritance models (codominant, dominant, recessive model, and log-additive), and the akaike information criterion (AIC) was considered for estimation of the relative value of statistical models. The level of significance was set up equal to 0.0083 (0.05/6 = 0.0083) based on Bonferroni correction for multiple comparisons. These analyses were carried out using the SNPassoc R library^43^. Haplotype estimation and association analysis for recurrent HPV dysplasia was performed using the *haplo*.*stats* R package. P-values were corrected by permutation after 10,000 permutations using the score test implemented in *haplo*.*stats*^44^. No formal sample size was calculated. The sample was defined as all HIV- infected with biopsy proven AIN2 or AIN-3, CIN2 or CIN3 confirmed by HRA or colposcopy.

## Supplementary information

Supplementary information
