# Supplementary material for: ADAR1 function affects HPV replication and is associated to recurrent human papillomavirus-induced dysplasia in HIV coinfected individuals
Source: Sci Rep. 2019 Dec 27;9:19848. doi: 10.1038/s41598-019-56422-x (PMC6934649; doi:10.1038/s41598-019-56422-x)

Supplementary information

ADAR1 function affects HPV replication and is associated to recurrent human papillomavirus-induced dysplasia in HIV coinfected individuals

Maria Pujantell, Roger Badia, Iván Galván-Femenía, Edurne Garcia-Vidal, Rafael de Cid, Carmen Alcalde, Antonio Tarrats, Marta Piñol, Francesc Garcia, Ana M. Chamorro, Boris Revollo, Sebastian Videla, David Parés, Javier Corral, Cristina Tural, Guillem Sirera, José A. Esté, Ester Ballana, Eva Riveira-Muñoz

Supplementary Table 1. SNP and genotype information.

SNP	Gene	Chromosome Position	HW p	% Genotyped	MAF
rs6699729 (A/T)	ADAR1	154610420	0.932	100	0.410
rs3766927 (C/T)	ADAR1	154591665	0.726	99.4	0.344
rs3766925(T/A)	ADAR1	154592236	0.889	100	0.306
rs3766924 (C/T)	ADAR1	154599350	0.003	100	0.191
rs9616 (A/T)	ADAR1	154583257	0.081	100	0.275
rs9427097 (T/G)	ADAR1	154583257	0.208	100	0.173

HW: Hardy-Weinberg; MAF: minor allele frequency.

Supplementary Figure 1

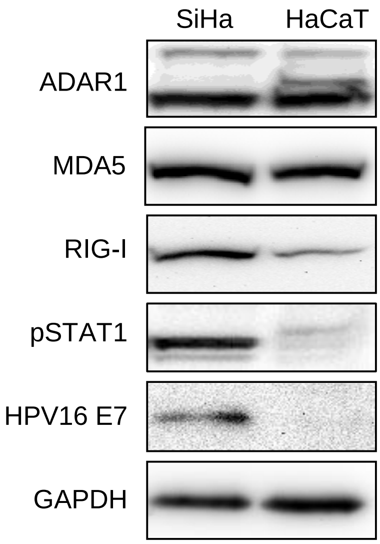

Supplementary Figure 1. Protein expression profile of players in the innate immune response between SiHa and HaCaT cell lines. Western blot of lysates from SiHa (HPV+) and HaCaT (HPV-) cell lines shows higher protein expression of RIG-I and phosphorylated STAT1 in SiHa (HPV16+) compared to HaCaT (HPV16-) cell line. Expression of MDA5, another cytoplasmic RNA sensor, and ADAR1 remained similar between SiHa and HaCaT. HPV16 E7 protein is only present in SiHa cell line, as expected. A representative western blot is shown.

Supplementary Figure 2

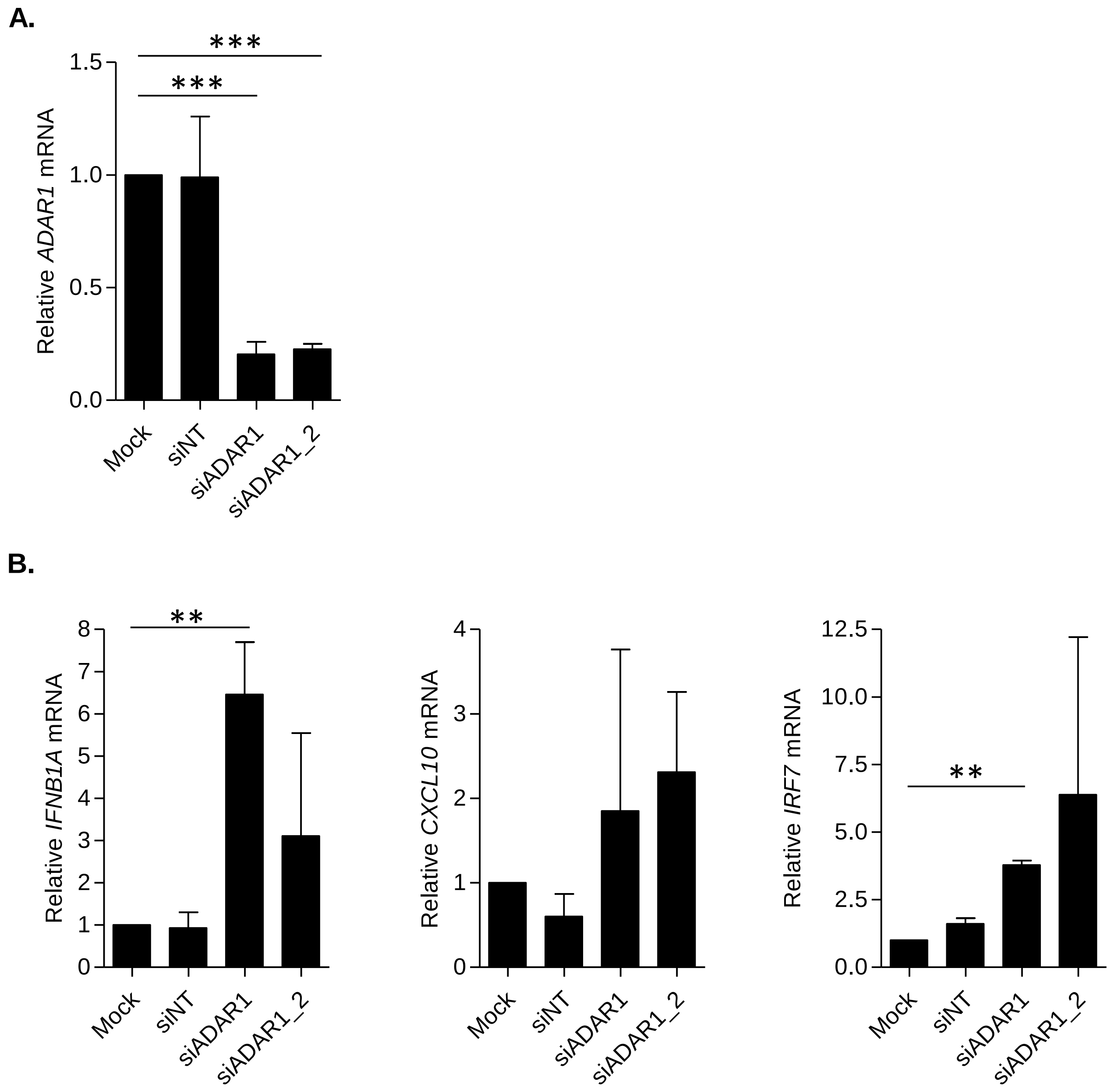

Supplementary figure 2. ADAR1 knockdown negatively regulates expression of IFN type I and RLR/MAVS signaling pathway mediators in SiHa cell line. (A) Effective downregulation of ADAR1 by two different siRNA in SiHa cell line. Relative mRNA expression of ADAR1 was measured by quantitative PCR and normalized to GAPDH expression. (B) Relative expression of INFB1A, CXCL10 and IRF7 in siADAR1 SiHa cells. mRNA expression was measured by quantitative PCR and normalized to GAPDH expression. INFB1A, CXCL10 and IRF7 gene expression was upregulated in siADAR1, whereas expression level in Mock-transfected or siNT did not change in SiHa cell line. Data represents mean ± SD of 2 independent experiments and is normalized to Mock-transfected SiHa cells. **p<0.005; ***p<0.0005.

Supplementary Figure 3

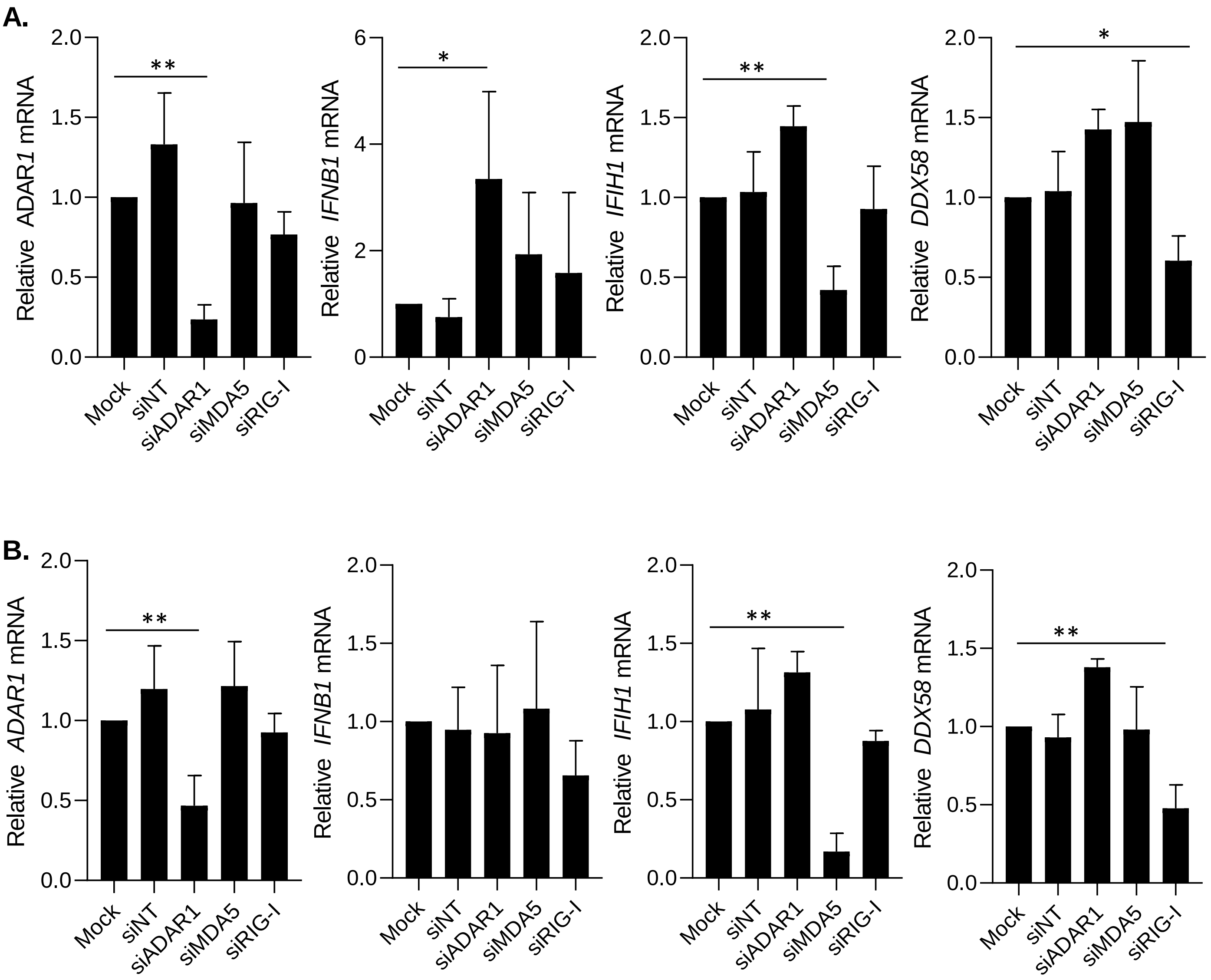

Supplementary figure 3. ADAR1 knockdown induces expression of IFN type I in RIG-I competent cells. ADAR1 expression was downregulated in Huh7 cells (A) and its derivative cell line Huh-7.5 (B), whose genome encodes a signaling-incompetent endogenous RIG-I (J. Virol. 2005;79:2689–2699). Upregulation of IFN expression in ADAR1 knockdown cells was only seen in Huh 7 cells that harbor a functional RIG-I. Only slight changes in IFN production were observed when RLR, MDA5 and RIG-I, were downregulated in Huh7 cells, whereas no change was seen in the signaling-incompetent RIG-I Huh7.5 cells. Moreover, ADAR1 knockdown was able to significantly increase RLR expression in Huh7 cells, further confirming that ADAR1 function modulates IFN expression through RLR signaling pathway. Gene expression was assessed by quantitative PCR and normalized to GAPDH expression. Data represents mean ± SD of at least 3 independent experiments and is normalized to Mock-transfected cells. siNT, non-targeting siRNA; *p<0.05; **p<0.005; ns, not-significant.

Supplementary Figure 4

A

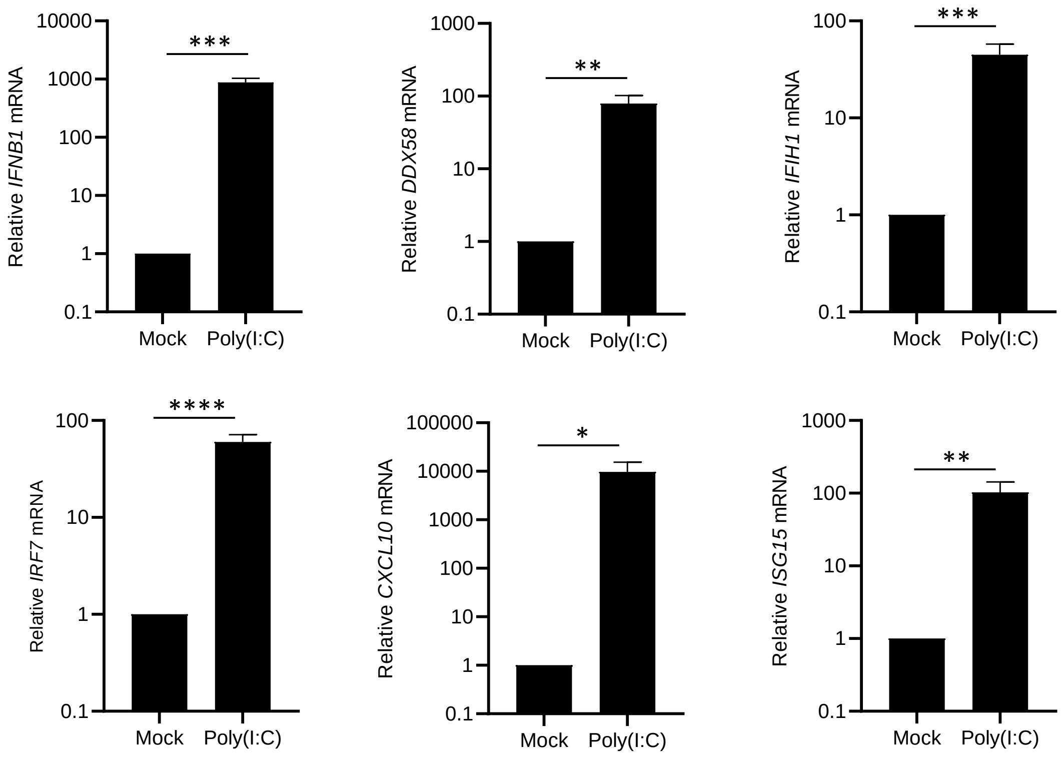

B

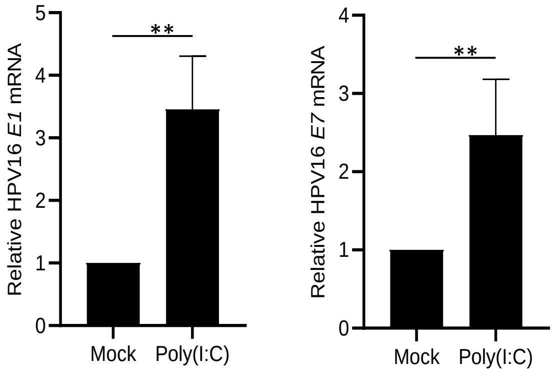

Supplementary figure 4. Poly(I:C) induces innate immune activation and enhances expression of HPV16 E1 and E7 genes in HPV+ cell line. Relative mRNA expression of (A) interferon stimulated genes (*IFNB1, DDX58, IFIH1 IRF7, CXCL10 and ISG15)* and (B) HPV16 *E1* and HPV16 *E7* genes, 16h post-transfection with 2µg of poly(I:C) per 1,25 x10^5 SiHa cells. Gene expression was assessed by quantitative PCR and normalized to GAPDH expression. Data represents mean ± SD of at least 3 independent experiments and is normalized to Mock-transfected cells.*$p<0.05$; **$p<0.005$; ***$p<0.0005$; ****$p<0.0001$.

Supplementary Figure 5

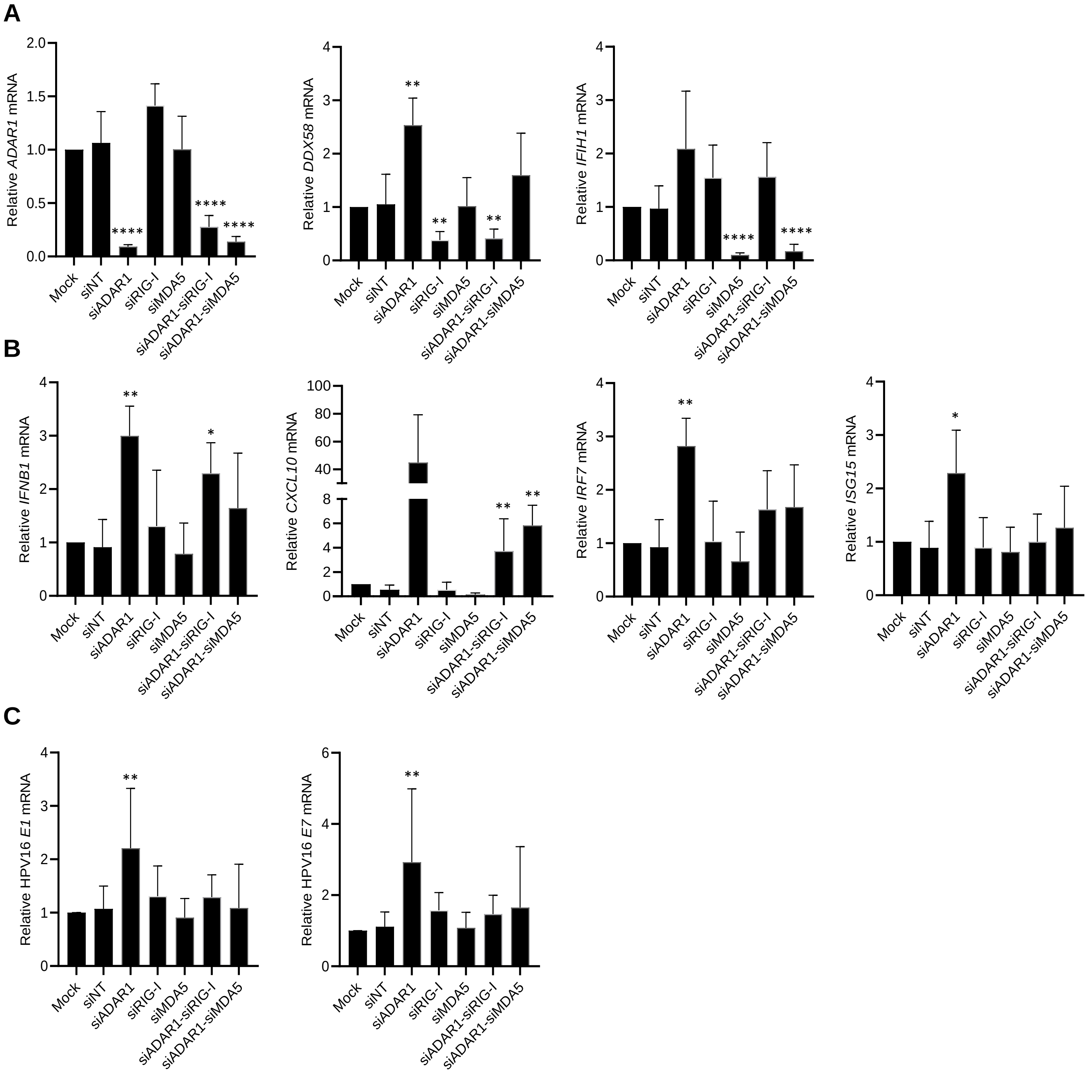

Supplementary figure 5. Gene expression profile of ADAR1, RIG-I and MDA5 knockdowns and ADAR1-RIG-I and ADAR1-MDA5 double knockdowns. (A) Significant downregulation of *ADAR1*, RIG-I (*DDX58)* and MDA5 (*IFIH1*) in all conditions. (B) Significant increase in gene expression of interferon stimulated genes (*IFNB1, CXCL10, IRF7 and ISG15)* in siRNA-ADAR1 knockdown cells, enhanced *IFNB1* in siADAR1-siRIG-I double knockdown and increase *CXCL10* expression in siADAR1-siRIG-I and siADAR1-siMDA5 double knockdowns. (C) Significant increase in gene expression of HPV16 *E1* and HPV16 *E7* only in siRNA-ADAR1 knockdown. 64h post-transfection with 50 pmol of siRNA, or 25 pmol of each siRNA for double knockdown, per 1,25 x10^5 SiHa cells. Gene expression was assessed by quantitative PCR and normalized to GAPDH expression. Data represents mean ± SD of at least 3 independent experiments and is normalized to Mock-transfected cells. *p<0.05; **p<0.005; ***p<0.0005; ****p<0.0001.

Supplementary Western Blot Images

Full-length Western blot to Fig. 1B: HaCaT cell line

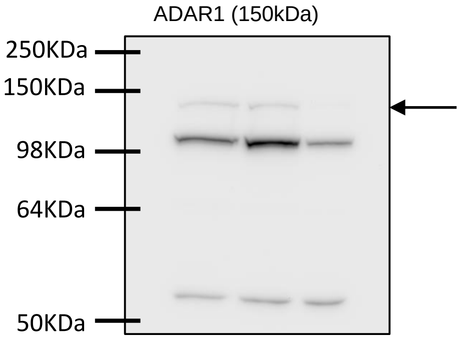

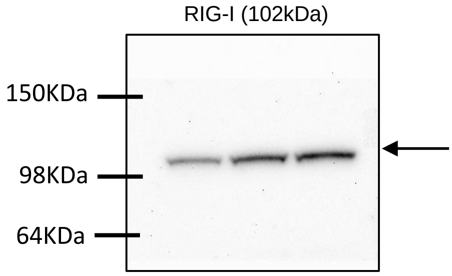

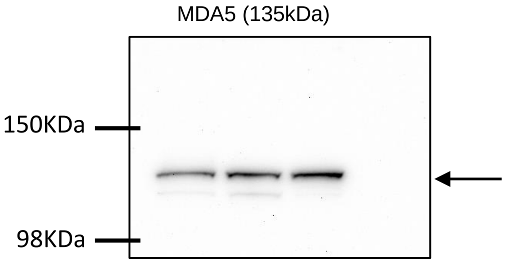

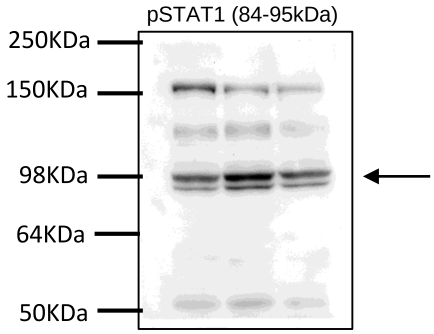

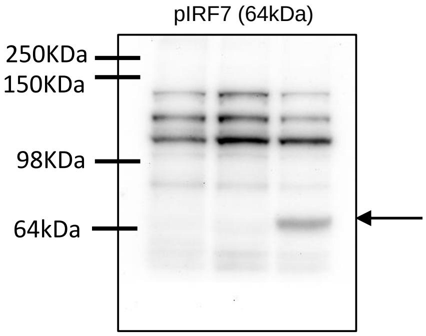

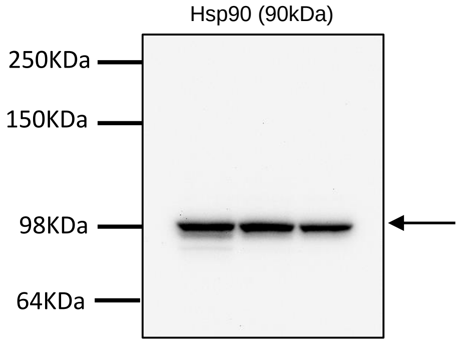

Full-length Western Blot to Fig. 1B: SiHa cell line

ADAR1 (150kDa)

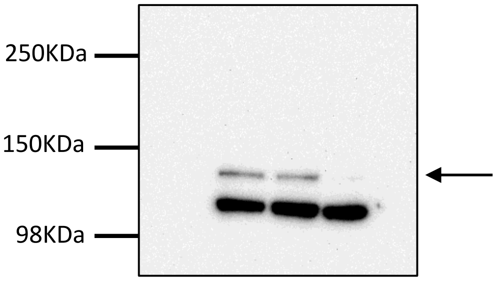

RIG-I (102kDa)

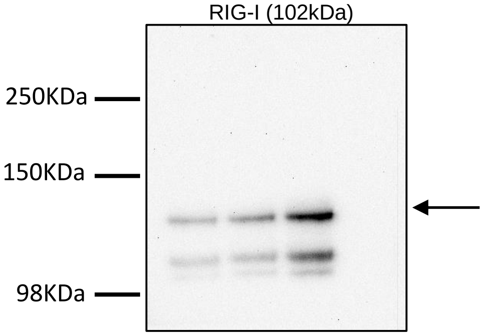

MDA5 (135kDa)

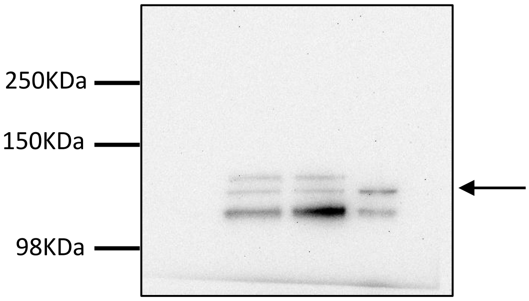

pSTAT1 (84-95kDa)

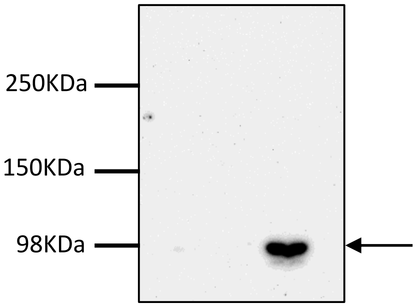

pIRF7 (64kDa)

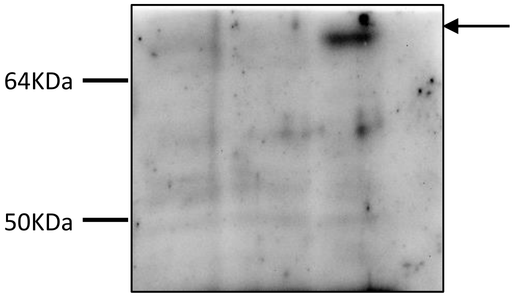

Hsp90 (90kDa)

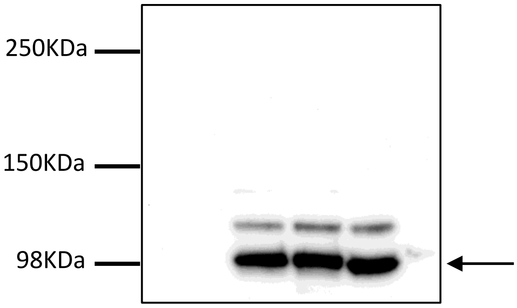

Full-length Western Blot to Fig. 3B

ADAR1 (150kDa)

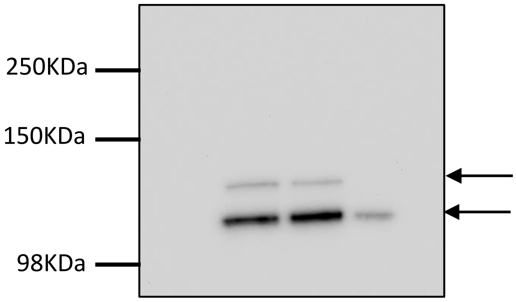

HPV16 E7 (17kDa)

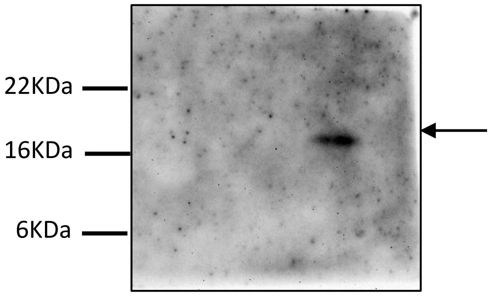

GAPDH (40kDa)

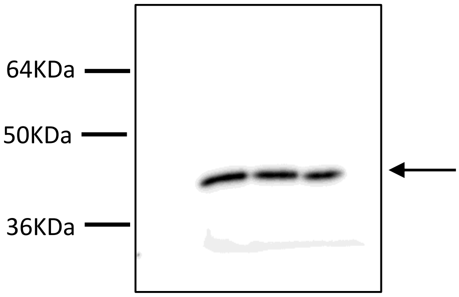

Full-length Western blot to Supp. Fig. 1B

ADAR1 (150kDa)

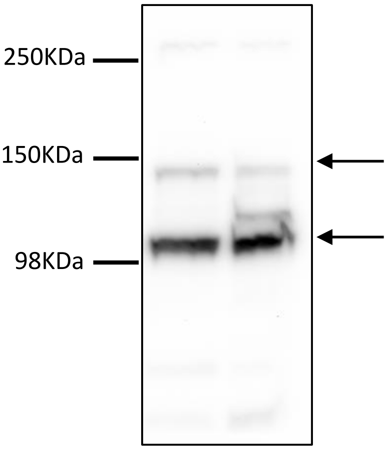

MDA5 (135kDa)

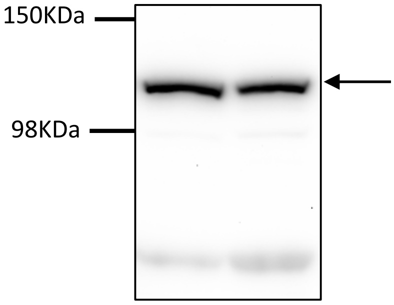

RIG-I (102kDa)

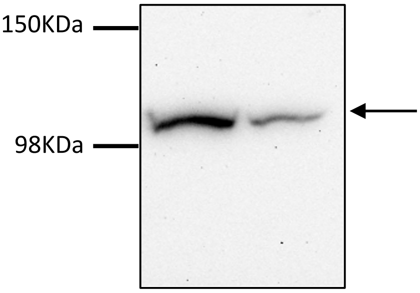

pSTAT1 (84-91kDa)

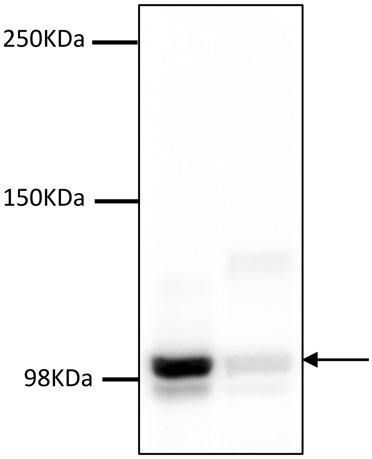

HPV16 E7 (17kDa)

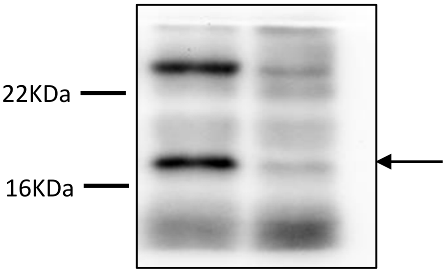

GAPDH (40kDa)

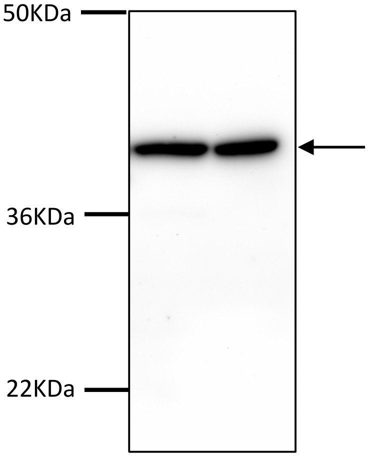

Supplement: Supplementary file 1 — Supplementary information [file 41598_2019_56422_MOESM1_ESM.pdf]
